# High EZH2 expression is correlated to metastatic disease in pediatric soft tissue sarcomas

**DOI:** 10.1186/s12935-016-0338-x

**Published:** 2016-07-28

**Authors:** Maria Ramaglia, Velia D’Angelo, Adriana Iannotta, Daniela Di Pinto, Elvira Pota, Maria Carmen Affinita, Vittoria Donofrio, Maria Elena Errico, Angela Lombardi, Cristiana Indolfi, Fiorina Casale, Michele Caraglia

**Affiliations:** 1 Department of Woman, Child and General and Specialized Surgery, Pediatric Oncology Unit, Second University of Naples, Via L. De Crecchio, 2, 80138 Naples, Italy; 2 Pediatric Pathology Unit, Pausilipon Hospital, Naples, Italy; 3 Department of Biochemistry, Biophysics and General Pathology, Second University of Naples, Naples, Italy

**Keywords:** EZH2, PRC2, Pediatric sarcoma, Polycomb proteins

## Abstract

**Background:**

Enhancer of Zeste *Drosophila* Homologue 2 (EZH2) is a key regulator of transcription as a member of polycomb repressive complex 2 (PRC2) which exerts repression of downstream genes and is correlated to invasiveness and progression of different tumours. Therefore, we evaluated the expression of PRC2 proteins in pediatric soft tissue sarcoma (rhabdomyosarcoma, RMS and extraosseous Ewing sarcoma, EES) correlating them to the clinical outcome of the patients.

**Methods:**

We analyzed PRC2 protein expression by quantitative real time PCR, western blotting and immunohistochemistry in 17 soft tissue sarcomas (11 RMS and 6 EES) enrolled at Paediatric Oncology Units of the Second University of Naples. Expression analysis was performed for EZH2, SUZ12 and EED.

**Results:**

Enhancer of Zeste *Drosophila* Homologue 2 was expressed with a different degree in 60 % of samples. Interestingly, the magnitude of EZH2 up regulation was significantly higher in patients presenting lymph node and/or distant metastases at the diagnosis. Moreover, patients overexpressing EZH2 had a lower probability of survival compared to patients negative or with low EZH2 expression.

**Conclusions:**

Our study suggests that high EZH2 expression is associated to increased aggressiveness of the disease. Therefore, drugs that control its activity could be potentially used in the epigenetic target treatment of tumors with these alterations.

## Background

The polycomb proteins retain the expression pattern of different cells by controlling chromatin structure during early development. In mammalians two polycomb group complexes are described, polycomb repressive complex 1 (PRC1) and 2 (PRC2). PRC2 complex, conserved from *Drosophila* to mammals, consists of Enhancer of Zeste *Drosophila* Homologue 2 (EZH2), Suppressor of Zeste (SUZ12) and Embryonic Ectoderm Development (EED).

Polycomb repressive complex 2 catalyses the methylation of lysine 27 at histone H3, a repressive mark, and is involved in many biological processes as differentiation, cell identity, proliferation and stem-cell plasticity [1].

Whether a tumor derives from an embryonic stem cell/multipotent progenitor or from the dedifferentiation of specialized cells, the initiation of malignant transformation can be also a result of epigenetic deregulation [2]. EZH2 is a histone methyltransferase that contains the SET domain with its active site. EZH2 overexpression was firstly linked to cancer by microarray analysis of prostate and breast cancer where it is associated to aggressive, metastatic disease and to a poor clinical outcome [3–7]. However, overexpression of EZH2 has also been described in other adult cancers including bladder, gastric, lung, and hepatocellular carcinoma [8].

Richter et al. [9] found that EZH2 can be induced by FLI1/EWS due to its binding to EZH2 promoter in Ewing sarcoma (ES) in vivo. In fact, about 85 % of ES are characterized by the specific translocation t(11;22)(q24;q12) inducing the specific transcript of the fusion protein FLI1/EWS. The correlation between EZH2 expression and growth control of ES was suggested by both ES growth inhibition in vitro and delayed tumor development and metastases in mice induced by RNA interference-mediated inhibition of EZH2. Moreover, down-regulation of EZH2 reduced nerve growth factor receptor (NGFR) expression, which is an essential marker of neuroectodermal stem cells. Mutations of EZH2 were also described in ES even if correlations to patient prognosis were not found [10]. In addition, epigenetic regulation of polycomb target genes, in particular HOXD genes, is altered in ES [11].

Enhancer of Zeste *Drosophila* Homologue 2 overexpression was also found in soft tissue sarcoma as it is a key factor in the proliferation and survival of PAX3-FOXO1 alveolar rhabdomyosarcoma (RMS) cells working, at least in part, by repressing FBXO32. In fact, the reduction of EZH2 activity represents a novel adjuvant strategy to eradicate high-risk PAX3-FOXO1 alveolar RMS [12].

It was also demonstrated that silencing of EZH2 promotes the recruitment of a multiprotein complex at muscle-specific promoters in RMS cells and is directly involved in the induction of a muscular differentiation program in these cells [13]. Some studies exist also on adult sarcomas such as synovial sarcomas in which it was found that high EZH2 expression is predictive of developing distant metastases even in the better-differentiated subtypes [14].On the other hand, poor data are available on childhood soft tissue sarcomas.

Epigenetic silencing of tumor suppressor genes, including EZH2, in cancer has evoked the use of potential therapeutic strategies based upon inhibitors of epigenetic enzymes. One reported anti-EZH2 drug is deazaneplanocin A (DZNep), an inhibitor of S-adenosyl-l-homocysteine (SAH) hydrolase that promotes degradation of the PRC2 complex and indirectly inhibits EZH2 through effects on intracellular SAH concentrations; GSK126 is a small-molecule that directly inhibits both wild-type and mutant EZH2 methyltransferase activities [15, 16].

Some studies showed that SUZ12 has been subjected to chromosomal aberrations in endometrial stromal sarcomas (ESS). EES account for less than 10 % of uterine sarcomas and the most frequent rearrangement is a t(7;17) translocation leading to the fusion of the JAZF1 and SUZ12 genes that map to chromosomes 7 and 17, respectively [17, 18].

While SUZ12 and EED mutations have only been found to have loss-of-function effects, EZH2 mutations have been found to have both activating and inactivating functions in different cancers [19].

Deletions and inactivating mutations of SUZ12 and EED are found in malignant peripheral nerve sheath tumors (MPNSTs) [20].

SUZ12 inactivating mutations cause a decrease or loss of H3K27me3 and a reciprocal gain of H2K27 acetylation and H3K4me3, which enables active transcription factors to be recruited and the disease to progress [21]. SUZ12 is overexpressed in many human cancers, as ovarian cancer, mantle cell lymphoma or non-small cell lung cancer and breast cancer [22].

In the present manuscript, we have studied the expression of the polycomb complex proteins EZH2, SUZ12 and EED in paediatric soft tissue sarcomas correlating the expression with the clinical outcome of the patients.

## Methods

### Patients and tumor samples

Tumor samples were collected from 17 patients affected by paediatric soft tissue sarcomas (11 RMS and 6 EE) treated at Paediatric Oncology Unit of Second University of Naples.

Tumors were classified according to the Intergroup RMS Studies (IRS) grouping: stage I, tumor macroscopically and microscopically removed; stage II, macroscopic complete resection but microscopic residuals; stage III, macroscopic complete resection but microscopic residuals and lymph nodes affected and not removed; and stage IV, metastasis present or non-regional lymph nodes involved. Normal controls (bone tissue and muscle) were formalin fixed and paraffin-embedded at our Pathology Unit. The study was approved by regional ethical review board at the Second University of Naples and performed in compliance with the Helsinki Declaration. All patients were aware and gave written informed consent.

### RNA extraction and quantitative real-time PCR

Total RNA was extracted from cell cultures using TRI REAGENT (Molecular Research Center Inc., OH, USA) according to the manufacturer’s protocol. RNA from paraffin-embedded tissues was extracted with RNeasy FFPE kit (Invitrogen). The reactions were run on an ABI PRISM^®^7900HT Sequence Detection System; the cycling conditions were 10 min at +95 °C (initial denaturation) followed by 40 cycles of 15 s at +94 °C (denaturation) and 1 min at +68 °C (annealing/extension/data collection). Specific primers for human EZH2, SUZ12, and EED were designed (Table 1). In the first step, we determined the stability of a control gene (Beta-actin) for the normalization of the real-time PRC products. Assays were performed in triplicate. We used the 2^−ΔΔCt^ method to analyse the obtained data.

**Table 1:** Primer sequences for quantitative real time-polymerase chain reaction

Gene	Sense	Antisense
EZH2	5′cgcttttctgtaggcgatgt 3′	5′tgggtgttgcatgaaaagaa 3′
SUZ12	5′gggagactattcttgatgggaag3′	5′actgcaacgtaggtccctga 3′
EED	5′gaaattccatccaagagatcca 3′	5′tggatattccataatcgtaaagca3′
β-actin	5′gcgagaagatgacccagatc 3′	5′ggatagcacagcctggatag 3′

### Protein extraction and western blot analysis

Proteins were extracted using lysis-buffer with protease-inhibitors. Protein concentration was quantified using Bradford assay (Bio-Rad). 30 μg of protein was run on polyacrylamide gel and blotted onto PVDF membrane (Millipore, Marlborough, MA). Immunoblotting was performed using primary antibodies against EZH2 (C-1), SUZ12 (D-10) and EED (H-300) (Santacruz Biotechnology, INC) (1:500). Bands were visualized using a chemiluminescent system (ECL-Amersham). Quantity One 1-D analysis software (Biorad Laboratories) was used to estimate the intensity of each band. Results were normalized against the level of actin expression in each sample. It was obtained a range of arbitrary intensities of each bands from 0 to 190 %, with a median value of 70 %. We have selected intensity value higher than 70 % in order to quantify the expression of the proteins as high, instead different proteins lower or equal to 70 % were considered as low expression.

### Immunohistochemistry

Tissue section of 4 mm-thick were deparaffinised, rehydrated and washed in PBS. Sections were quenched sequentially in 3 % H_2_O_2_ for 15 min and blocked with PBS-6 % not fat-dry milk (Biorad; Hercules, CA) for 60 min at room temperature. EZH2 (ENX-1)-166,609 primary antibody (SantaCruz Biotecnology, INC) was incubated at 1:100 dilution at 4 °C overnight. Secondary anti-mouse biotinylated antibody was incubated at 1:200 at 4 °C for 60 min.

Automated autostainer (Dako Immunostainer) was utilized to visualize positive slides with avidin–biotin-peroxidase method. The percentage of positive nuclei stained for EZH2 was quantified by scoring for semi-quantitatively levels of expression. At least 5 fields per section by 2 blinded observers was considered; in rare cases of discrepancy, the sections were analyzed by an additional two independent observers. EZH2 was considered positive when nuclear staining was identified in at least 10 % of the neoplastic cells. On the other hand, normal lymphocytes were all immune negative for EZH2 in same sections. A negative control was obtained by replacing the primary antibody with a normal murine IgG. EZH2-staining intensity was scored weak when recorded in at least 10 % cells and strong when recorded in more than 10 % cells. Each section was observed using an DM LB2 microscope (Leica St. Gallen, Switzerland) at 400× magnification. Images were acquired through Digital Leica software with Leica Digital Camera DM LB2.

### Statistical analysis

The Chi squared test was used for univariate analysis of PRC2 proteins and categories of different prognostic factors. The patients’ dichotomous variables were assessed according to their ability to influence overall survival (OS). Survival differences (OS), estimated at 5 years by the Kaplan–Meier analysis, were evaluated using a log-rank test.

In the OS analysis, all deaths were considered treatment failures. Results were expressed as probability (percent) and 95 % confidence intervals (CI). Data are given as mean ± SD. Differences were assessed by *t* test and p < 0.05 were considered statistically significant.

## Results

### Main clinical and pathological characteristics of the patients

Seventeen paediatric patients (6 males and 11 females), affected by RMS or EES, were included in this study. The age of patients ranged from 3 to 160 months (median 55 months, mean: 71.7 ± 55.4). According to the IRS grouping 1 patient with RMS was classified as stage II, seven as stage III and three as stage IV, respectively. Among the 6 patients with EES 1 was stage II, 4 stage III and 2 stage IV, respectively (Table 2). Overall, at a 5-year follow-up (median = 60 months 95 % CI 18.3–60.0), 9 patients (52.9 %) were alive and in continuous complete remission.

**Table 2:** Clinical and biological data of the 17 patients enrolled in the study

Clinical characteristics	Number of patients (%)
Gender	
Males	6 (35.3)
Females	11(64.7)
Age(months)	
Minimum	3
Median	55
Maximum	160
Histopathology	
RMS	11 (64.7)
EES	6 (35.3)
Stage	
RMS and EES	
I	0
II	2 (11.7)
III	11 (64.7)
IV	4 (23.5)
Outcome	
Alive	9 (52.9)
DOD	8 (47)

*RMS* rhabdomyosarcoma; *EES* extraosseous Ewing sarcoma; *DOD* dead of disease

### EZH2 was highly expressed in cancer cells of soft tissue sarcomas (RMS and EES)

Many studies showed that expression of EZH2, the catalytic subunit of PRC2 complex, is frequently overexpressed in several adult tumors. In order to confirm these data in paediatric cancers, we analyzed gene and protein expression of PRC2 subunits.

The quantitative analysis of primary samples at diagnosis (11 RMS and 6 EES), based on RNA sample availability, showed an overexpression only of EZH2 but not of either EED or SUZ12 that showed expression levels similar to control samples and normal counterparts. In details, the analysis showed 0.2 to 4.1-fold higher expression of EZH2, compared to the control samples, in either RMS or EES, respectively (p = 0.02 and p = 0.008, respectively).

**Fig. 1:**
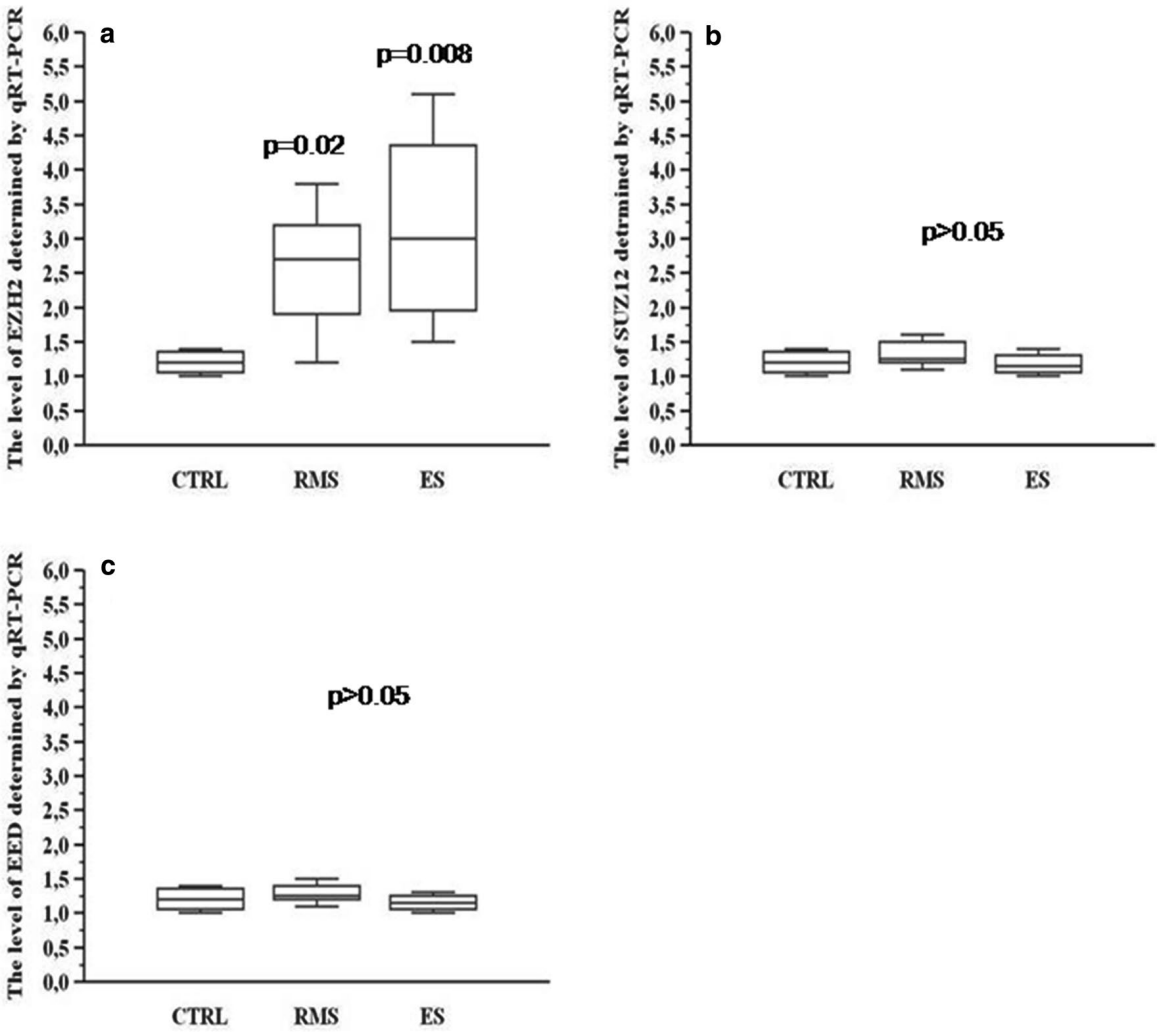
**a** EZH2, **b** SUZ12 and **c** EED expression evaluated through real time PCR between the control samples (CTRL) and soft tissue sarcoma samples

On the other hand, SUZ12 and EED expression levels were 0.2 to 1-fold and 0.07 to 1-fold decreased, respectively, if compared to normal controls (p > 0.05) (Fig. 1).

Moreover, we studied the protein expression of EZH2, SUZ12, and EED, by immunoblotting.

**Fig. 2:**
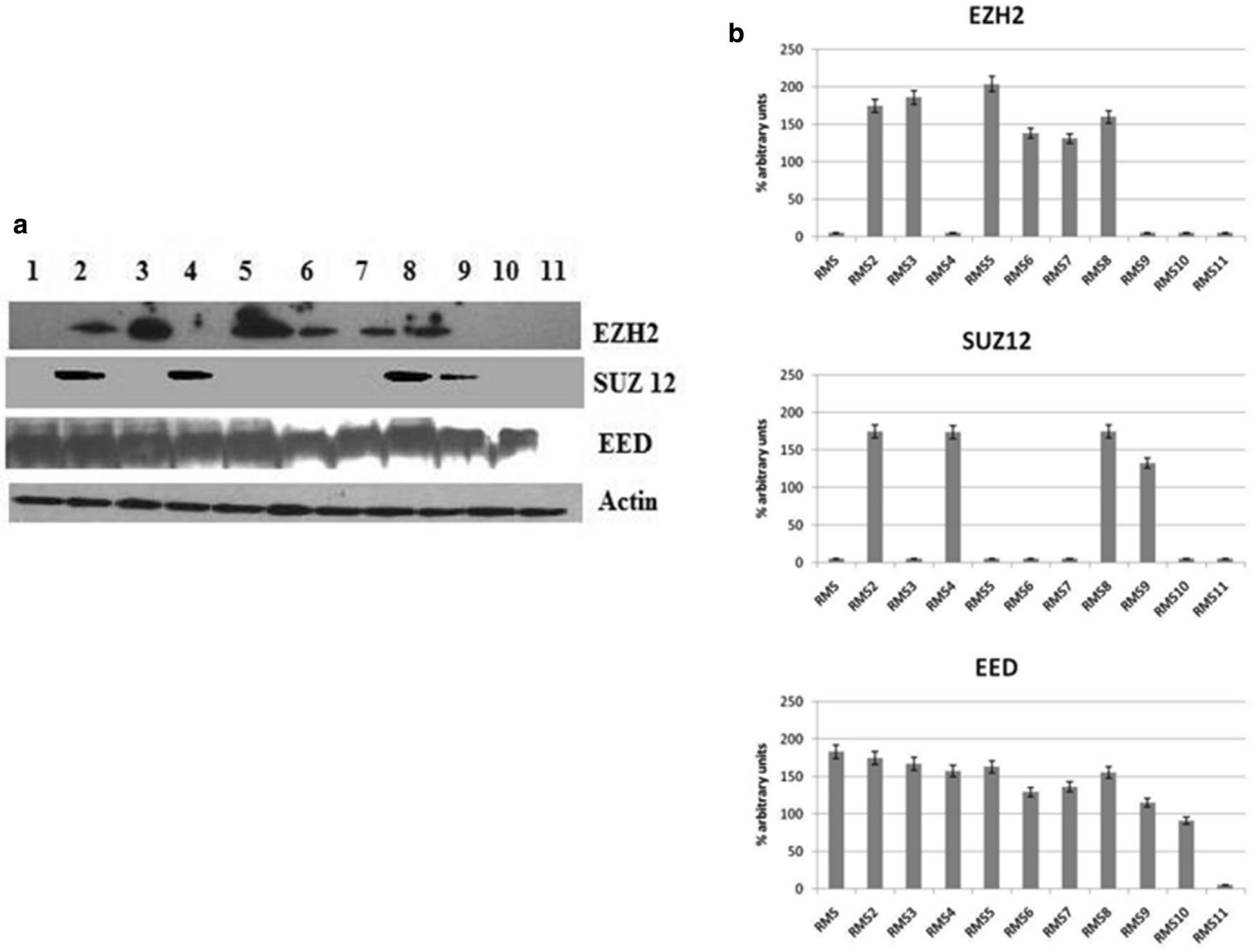
PRC2 expression (EZH2, SUZ12 and EED) in RMS samples evaluated through immunoblotting (**a**) and representation of the densities of the bands expressed as percentage of expression after laser scanning (**b**)

**Fig. 3:**
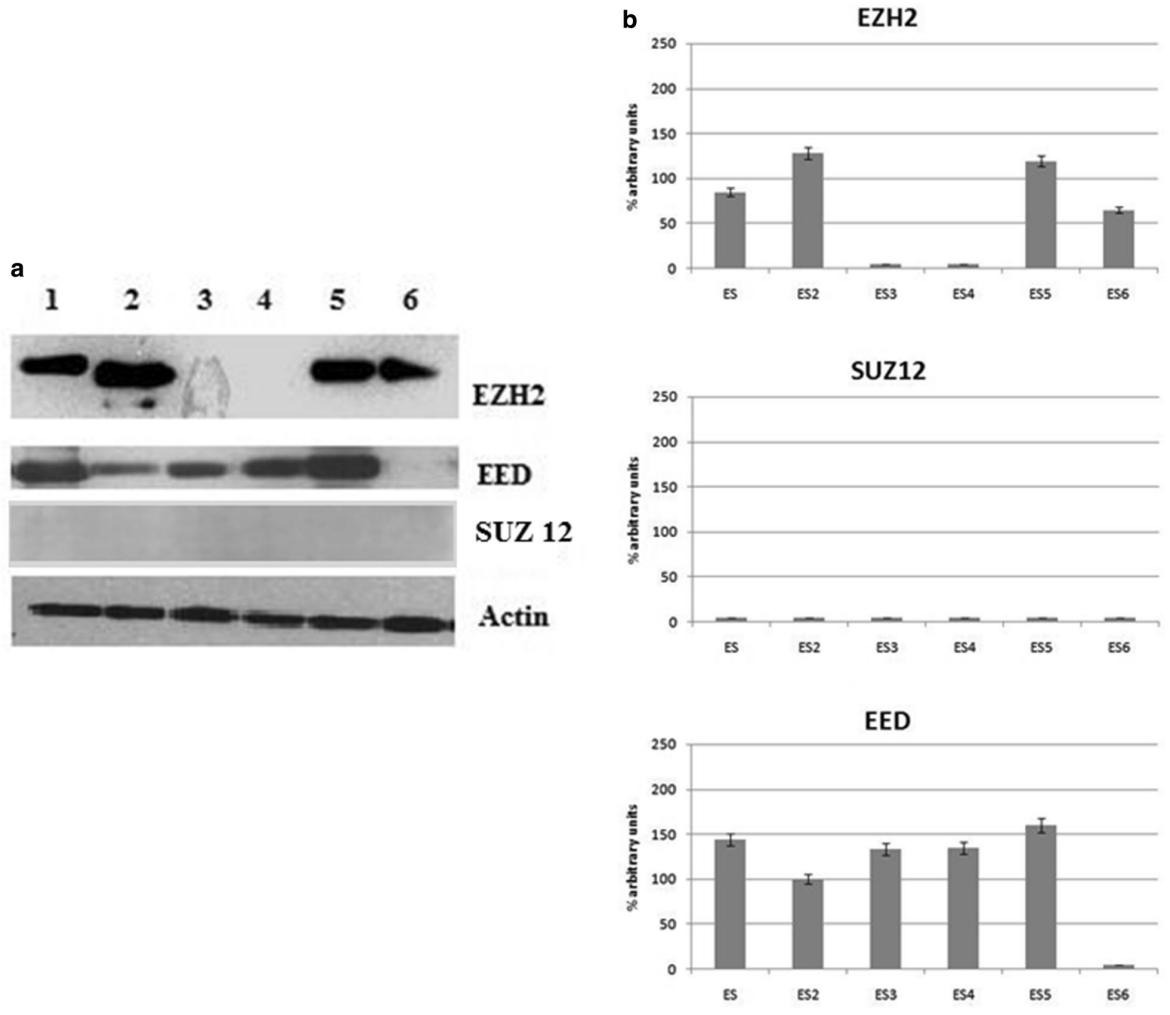
PRC2 expression (EZH2, SUZ12, and EED) in EES samples evaluated through immunoblotting (**a**) and representation of the densities of the bands expressed as percentage of expression after laser scanning (**b**)

We have observed protein expression in 10 samples (58.8 %) for EZH2, 4 (23.5 %) for SUZ12, and 15 (88.2 %) for EED out of the 17 patients enrolled in the study, respectively. Western blotting analysis suggested a general upregulation of EZH2 and totally confirmed the results obtained by qRT-PCR. We have also quantified proteins expression through the densities of the corresponding bands after laser scanning and represented the results as percentage. EZH2 showed low levels of expression in 2 samples (20 %), and high levels in the remaining 8 (80 %) out of the 10 samples analysed; the four SUZ12 positive samples had high expression, while EED showed low expression in 1 sample (7 %) and high expression in 14 (93 %) out of the 15 samples examined (Figs. 2, 3). When we compared EZH2, SUZ12 and EED expression with clinical and biological characteristics of patients, we did not find any significant correlation.

Immunohistochemical analysis was performed in primary tumors to quantify and localize the expression of EZH2 protein. Microscopy pictures showed nuclear localization of EZH2.

**Fig. 4:**
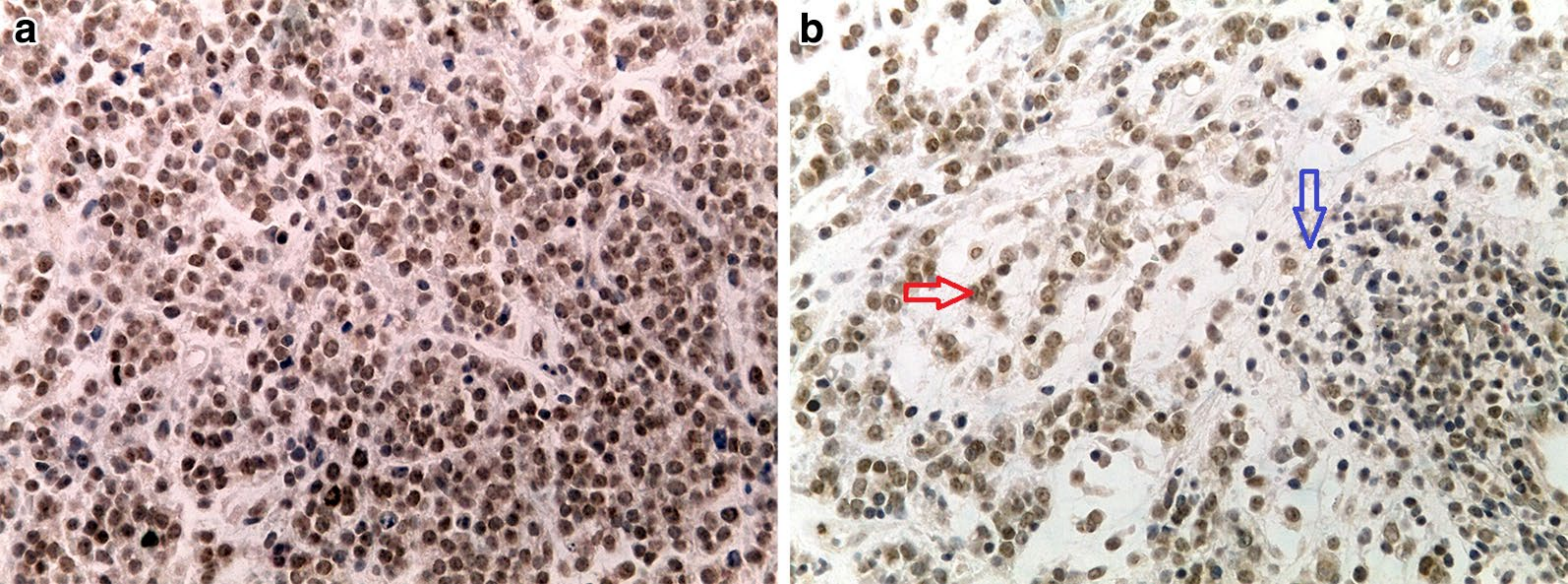
(**a**, **b**) Representative immunohistochemical staining showing EZH2 expression in sections of primary tumor tissue of RMS.* Brown-orange* color in nuclei indicates strong positive staining (400× Magnification). Normal control lymphocytes are negative. *Red arrow*: positive area; *blue arrow*: negative normal lymphocytes

Strikingly, nuclear EZH2 protein staining was low in 2/10 (Fig. 4a) and high (Fig. 4b) in 8/10 of analyzed positive samples. These findings confirmed that the expression of EZH2 protein is elevated in primary tumors. The data obtained with IHC had a good correlation with those obtained by western blotting (p < 0.05).

### EZH2 mRNA high levels are correlated to metastatic disease

Heterogeneity of molecular expression within individual tumors and between the localized and metastatic tumors has been reported in some types of cancers [23–25].

**Fig. 5:**
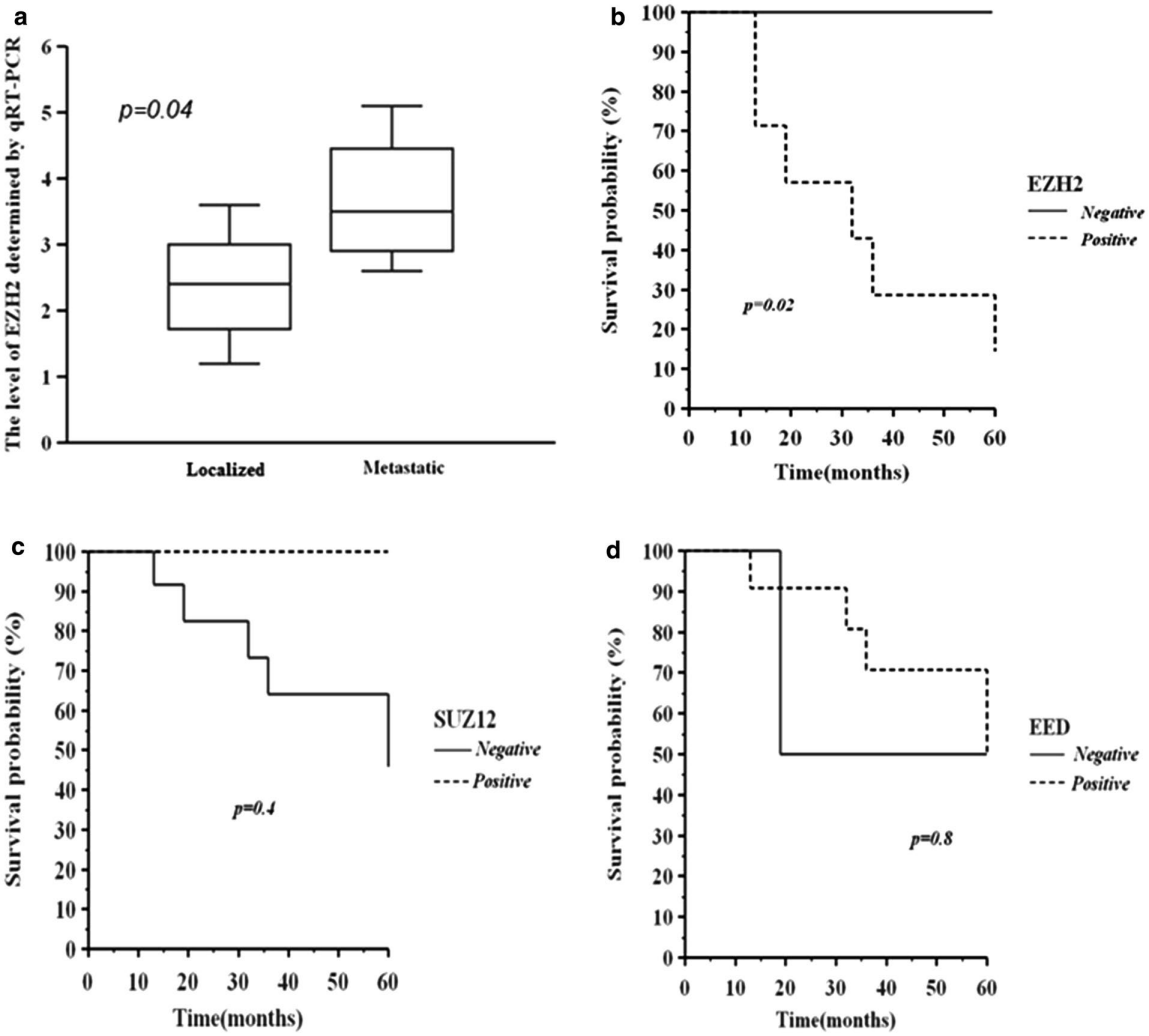
**a** EZH2 expression evaluated through real time PCR in metastatic and localized soft tissue samples. **b** All patients Kaplan–Meier analysis of overall survival (OS) stratified for EZH2, **c** SUZ12 and **d** EED expression

We grouped the patients on the basis of EZH2 high or low expression and presence or absence of regional/distant lymph node and/or distant metastases. The patients with intratumour EZH2 high expression had stage III (positive lymph nodes and not removed) or stage IV at diagnosis (8/17). In fact, the fold-change average of EZH2 in this group was 3.7, whereas in the other patients (9/17) was 2.2, close to the limit of 1.1-fold used for the detection of mRNA expression. The *t* test performed on the two groups with low and high EZH2 expression, suggested that higher EZH2 mRNA levels were correlated to presence of lymph node and/or distant metastases (p value = 0.04) (Fig. 5a).

The Kaplan–Meier analysis of all patients, comparing OS curves after 5 year from diagnosis, demonstrates that the 10 patients EZH2-expressing had a lower probability of survival compared to the 7 cases negative for EZH2 (15 vs 100 %) (log-rank test, p = 0.02, Fig. 5b). No significant differences in OS rate were found between patients stratified according to SUZ12 and EED expression (log-rank test p > 0.05) (Fig. 5 c and d).

## Discussions

Cancer is considered a developmental disease and changes of biologic processes, that are central to normal embryonic development, are main features of human malignancy. Nowhere, this is more evident than in paediatric tumors, where alterations of normal development are believed to be the origin of many childhood tumors. Over the past decade, to begin a new course for innovative anti-cancer treatments, pre-clinical studies on the modulation of epigenetic regulators have been performed. Since epigenetic processes are key players in cell tissue specification during the embryonal life, this approach seems to be particularly attractive for those cancers, such as pediatric cancer, in which the pathogenic mechanism implicates the deregulation of genes controlling the lineage commitment [2]. Many studies have shown that the overexpression of EZH2 in human cancers is often associated with poor prognosis [4, 12, 26]. Undifferentiated stem-like cancers express EZH2 that promotes cancer cell proliferation and metastasis formation by inhibiting several tumor suppressor genes [27].The present work was aimed to evaluate expression of PRC2 subunits in 17 paediatric tumor samples and to correlate them to the prognosis of the tumour diseases. In this study, we report that EZH2 is aberrantly over-expressed in primary tumors if compared to normal tissues, thus indicating that the high levels of expression of EZH2 is a common molecular lesion of RMS and EES. Moreover, we show that its expression is significantly correlated to a lower probability of OS. Interestingly, *t* test performed on the two sub-groups with low or high EZH2 expression, suggests that higher EZH2 mRNA levels are correlated to the presence of lymph node and/or distant metastases. The toleration and diffusion of genetic lesions that trigger malignant transformation depends upon epigenetic plasticity in the target cell. This model is longer supported in paediatric solid tumors that, at the contrary of adult malignancies, derive from relatively few genetic mutations progressing to invasive, drug-resistant and metastatic phenotypes in spite of the presence of rather stable genomes [2]. Dysregulation of EZH2 might confer acquired proliferative and migratory properties reverting cells that normally differentiate. Moreover, high expression levels of EZH2 detected in primary tumor tissue could be exploited to improve quality of tumor stage classification. Our results agreed with Richter and colleagues that found EZH2 responsible for the undifferentiated phenotype of EES by maintaining a stemness gene expression signature, inhibiting differentiation [9]. EWS/FLI1 has been found to induce the expression of EZH2 by direct binding to its promoter in ES cell lines. EZH2 expression and function were regulated by numerous different mechanisms, which could lead to the overexpression or increased activity of EZH2 in RMS. The involvement of the MYC transcription factor family, particularly MYCN, has been showed in RMS tumorigenesis. Interestingly MYC induces EZH2 expression by repression of microRNA-26a [28]. Also, EZH2 expression is induced by E2F1 and repressed by activated Rb, and its activity is stimulated by CDK1/2.

The particular association of characteristic fusion genes with RMS and EES suggests that chimeric oncoproteins play a leading role in the development of these paediatric sarcomas. In both entities, expression of aberrant fusion proteins affects important transcriptional regulators and promotes cellular transformation by modulating the transcription of specific target genes [29]. Our results were in agree with Burdach and colleagues that demonstrated loss of regulation of both EED and SUZ12 expression in Ewing tumors [30].

We hypothesize that the different expression between RNA and protein levels could depend on post-translational regulation for these two genes, by mutation in ubiquitin system or in its targets resulting in proteins stabilization; in a next study we will extend in a larger sample size the evaluation of mRNA and protein expression of these epigenetic regulating factors and we will analyze the mechanisms at the basis of the different expression of their mRNAs and proteins.

In conclusion, our data provide evidence that EZH2 abnormal over-expression is involved in sustaining proliferation and inhibiting myogenic differentiation of paediatric soft tissue sarcomas. More importantly, our results indicate that pharmacological targeting of EZH2 might represent a potentially valid approach to be used as treatment adjunctive to conventional therapy in order to make the latter more efficacious. Although there is an obvious rationale for inhibiting EZH2 function in malignancies in which this gene is overactivated, the finding of inactivating mutations in myeloid neoplasms suggests that this approach will need to be employed with caution. Understanding how to effectively combine current treatments with future histone demethylating agents will be a major challenge in the coming years.

## Conclusions

Our study suggests that unfavourable outcome and tumor progression of paediatric soft tissue sarcoma is linked to abnormal over-expression of EZH2 and malignancy grade is its effect. Novel therapeutic strategies in the inhibition of carcinogenesis and progression of paediatric could be provided by EZH2 inhibition.
